# The moderating effect of mental toughness on the relationship between stress and mental health

**DOI:** 10.3389/fpsyg.2026.1768455

**Published:** 2026-07-01

**Authors:** Anna Dziuba, Fabienne Ennigkeit, Janina Krell-Roesch, Larissa Appel, Chris Englert, Alexander Woll

**Affiliations:** 1 Department of Sports Sciences, Goethe University Frankfurt, Frankfurt, Germany; 2 Institute of Sports and Sports Science, Karlsruhe Institute of Technology, Karlsruhe, Germany

**Keywords:** depressive symptoms, mental health, mental toughness, resilience, stress, well-being

## Abstract

**Introduction:**

Mental toughness (MT) is increasingly recognized as a psychological resilience factor that can help individuals to cope with stress more effectively and maintain better mental health. While previous studies suggest that MT buffers the negative effects of stress, it is unclear whether these patterns are consistent across broader adult populations and multiple dimensions of mental health.

**Methods:**

This preregistered study examined whether MT moderates the relationship between perceived stress and three indicators of mental health: depressive symptoms, the mental health component of the SF-12 and subjective well-being. Participants were 402 community dwelling adults (aged 34–90) taking part in the longitudinal ‘Gesundheit zum Mitmachen’ project. MT, perceived stress, depressive symptoms, the mental health component, and subjective well-being were assessed using validated self-report instruments. Bivariate correlations and moderated regression analyses performed with Hayes’ PROCESS macro for SPSS were conducted.

**Results:**

Higher levels of MT were associated with lower perceived stress and more favorable mental health outcomes, including fewer depressive symptoms, higher mental health component and higher subjective well-being. Perceived stress was strongly related to poorer mental health across all indicators. MT significantly moderated the relationship between stress and both depressive symptoms and the mental health component. However, the interaction term did not reach significance for subjective well-being, although the descriptive pattern suggested a similar buffering tendency.

**Conclusion:**

The findings suggest that MT may be important in understanding individual differences in the association between perceived stress and mental health among community-dwelling adults. The observed pattern of results further supports the notion that the role of MT may vary across different mental health indicators rather than exerting uniform effects across outcomes. This is consistent with the view of MT as a context-dependent resource in stress appraisal and coping processes. Given the cross-sectional study design, the findings should be interpreted as associative rather than causal and require confirmation in longitudinal and experimental research.

## 1 Introduction

Mental health represents a fundamental component of overall health and well-being (Keyes, 2002). In line with contemporary public-health frameworks, mental health can be described as a dynamic state that enables individuals to cope effectively with life’s challenges, realize their potential, work productively, and participate meaningfully in society (World Health Organization, 2022). Rather than reflecting a simple absence of illness, mental health spans on a continuum ranging from optimal psychological functioning and well-being to severe emotional stress and psychological impairment (Antonovsky, 1996). Consequently, it holds both intrinsic value for individual flourishing and instrumental importance for social participation.

Psychological stress constitutes an important factor in mental health. Both modern society in general and sport environments in particular are often characterized by high psychosocial demands (e.g., pressure to perform, time constrains, and uncertainty) that increase the likelihood of stress experiences (Hanton et al., 2005). The transactional model of stress and coping developed by Lazarus and Folkman (1984) provides a well-established theoretical framework for understanding stress reactions. In contrast to earlier stress theories, Lazarus and Folkman assumed that it is not the (objective) nature of the stimuli or situations themselves that are triggering the stress reaction, but rather the (subjective) appraisal of these stimuli or situations and the coping resources of the affected individual. According to Lazarus and Launier (1978), stress is not a characteristic of the situation itself, but rather the result of a dynamic interplay between situational demands of the environment and personal resources to cope with these demands. When individuals perceive demands as important and manageable, they may view the situation as a challenge, leading to positive, motivating stress responses (eustress). However, when demands are perceived as exceeding available resources, the situation is experienced as a threat, increasing the likelihood of negative stress reactions (distress).

This perspective is particularly relevant in the field of sport and exercise, as athletes are often confronted with situations that are perceived as being either challenges or threats, depending on their subjective cognitive appraisal. In the primary appraisal, athletes first assess whether an event involves potential gains, losses, or risks. In a secondary appraisal, they assess their perceived control, coping options, and personal resources. These appraisal processes influence emotional and behavioral responses to competition, training stress, or social pressure and ultimately determine both performance and psychological well-being (Nicholls et al., 2016). Negative effects on mental health are more likely when the balance between the environment and the individual is unfavorable over a longer period of time (Hobfoll, 1998). Since appraisal is central to the stress response, stable personal resources play a crucial role in how individuals interpret and respond to challenging situations. Personal resources such as mental toughness (MT) can influence whether individuals view high-pressure situations as manageable challenges or overwhelming threats.

MT can be considered as both as a stable trait and a more dynamic, state-like resource that evolves with experience (Gucciardi, 2020). Originally conceptualized within elite sport as a key determinant of athletic performance (Clough and Strycharczyk, 2012; Jones et al., 2007), nowadays, MT is considered a broader psychological characteristic which helps individuals to perform well under stress (Crust, 2008). Higher levels of MT have been linked to higher self-beliefs, emotional control, persistence, and confidence in potentially challenging situations (Gucciardi, 2017; Lin et al., 2017). MT is assumed to influence both the primary and secondary appraisal of stressors, making them appear more controllable or meaningful, and serving as a coping resource. Thus, MT influences stress intensity and may protect against stress-induced declines in mental health. Furthermore, MT also influences the selection of specific coping strategies (Kaiseler et al., 2009).

The present study is based on Clough et al.’s (2002) 6C model of MT, which extends Kobasa’s (1979) concept of hardiness. Hardiness describes a resilient disposition characterized by three key components, i.e., control, commitment, and challenge, reflecting an individual’s ability to withstand and adapt to adversity (Maddi, 2004, 2006). Clough et al.’s (2002) conceptualization of MT added confidence as additional component. Within this framework, two of the four key components are further differentiated into two subdimensions, resulting in the so-called 6Cs. Control comprises the subdimensions life control and emotional control, whereas confidence is further differentiated into confidence in abilities and interpersonal confidence. While control refers to the perceived ability to influence life circumstances and regulate emotions, commitment reflects a proactive engagement with tasks and goals instead of passively accepting a given situation. Furthermore, challenge describes the tendency to perceive change as an opportunity for growth rather than a threat. Confidence refers to the belief in one’s abilities and interpersonal skills to overcome problems (for more information on the conceptualization of MT, see Dziuba et al., 2025a).

According to the 6C model (Clough et al., 2002), mentally tough individuals tend to view their personal environment as controllable, maintain strong self-belief, remain focused under pressure, and see difficulties as opportunity for growth (Jones et al., 2002). These characteristics contribute to more adaptive stress appraisals and higher stress tolerance (Crust and Clough, 2005). Moreover, higher levels of MT have been shown to be associated with athletes experiencing less stress and having more control over a self-selected sport stressor (Kaiseler et al., 2009). It has also been found that mentally tough individuals tend to use problem-focused coping strategies rather than avoidance strategies (Crust and Azadi, 2010; Kaiseler et al., 2009; Nicholls et al., 2008) and to possess more internal and external resources (e.g., positive identity, support, and empowerment) that are theorized to promote thriving and healthy psychosocial development (Gucciardi and Jones, 2012). In contrast, individuals with lower levels of MT tend to perform worse after negative feedback, suggesting reduced emotional robustness (Clough et al., 2002). While mentally tough athletes experience similar emotional intensity to others, they seem to adapt to these emotions more effectively (Crust, 2009; Gucciardi et al., 2017).

Although MT has primarily been used to explain individual differences in sport performance, research increasingly highlights its relevance for general mental health and psychological resilience in both athletes and the general population (Clough and Strycharczyk, 2012; Nicholls et al., 2009). In order to capture the complexity of mental health, this study focuses on three different indicators: depressive symptoms, the mental health component, and subjective well-being. This selection is based on the idea that mental health is a complex concept involving both psychological distress and positive functioning, as well as broader aspects of quality of life. Depressive symptoms and subjective well-being are affective-cognitive indicators that can be situated at opposite ends of a mental health continuum. However, in line with dual-continuum perspectives, they are not considered to be strict opposites, but rather as related yet partially independent aspects of mental health (Keyes, 2002; Westerhof and Keyes, 2010). For each of these indicators, significant relationships with MT has been demonstrated.

Depressive symptoms are an important indicator of mental illness and have been shown to be inversely related to MT in various population groups. For example, research in high school students, vocational students, and university students indicates that those with higher levels of MT reported fewer symptoms of depression and burnout when exposed to high stress compared to individuals with lower levels of MT (Gerber et al., 2013a; Gerber et al., 2013b; Gerber et al., 2015). Similar findings among elite athletes demonstrated that MT buffers the negative consequences of stress exposure on mental health, both cross-sectionally and prospectively (Gerber et al., 2018). Madigan and Nicholls (2017) further showed that higher levels of MT predicted decreases in burnout symptoms over time among junior athletes, and thus might act as a buffer against burnout-related symptoms in sports. These findings are consistent with the theoretical assumption that the four components of MT counteract the cognitive patterns characteristic of depression and burn-out.

The mental health component was included as a broad indicator of psychological functioning and mental health-related quality of life, capturing both emotional and functional aspects of well-being (Ware et al., 1996). MT has also been empirically linked to higher levels of emotional stability, fewer psychological complaints, and better overall psychological functioning (Lin et al., 2017; Papageorgiou et al., 2018). These associations suggest that MT leads to more adaptive appraisal processes and more efficient coping.

Subjective well-being, reflecting positive affect and life satisfaction, represents the positive end of the health continuum. Mentally tough individuals tend to report higher levels of subjective well-being and life satisfaction (Clough et al., 2002; Lin et al., 2017; Gerber et al., 2013a). Such findings are consistent with the notion that mentally tough individuals interpret stressors as manageable and maintain engaged in goal-directed behavior, which may foster better performance (Kaiseler et al., 2009).

While previous studies, particularly those by Gerber et al. (2015), Gerber et al. (2018), Gerber et al. (2013a), and Gerber et al. (2013b), have provided important evidence that MT can buffer negative stress effects, several gaps remain. First, the work by Gerber et al. has largely focused on adolescents, students, or elite athletes, making it less clear whether these findings are generalizable to more heterogeneous samples including both athletes and non-athletes across a wider age range. Second, many studies have examined single mental health outcomes, even though mental health comprises multiple distinct yet related dimensions. To address these gaps, the present study has two specific aims: (1) To examine associations between MT, perceived stress, and different facets of mental health, i.e., depressive symptoms as a marker of mental ill-health, mental health component as a broader indicator of psychological functioning and vitality (Ware et al., 1996), and subjective well-being as a positive, affective–evaluative indicator of mental health; and (2) to test the extent to which MT moderates the negative relationship between stress and mental-health outcomes in a large and diverse community-based sample of adults. Based on prior evidence, the following two hypotheses were formulated:

*H_1_*: MT is negatively correlated with (a) perceived stress and (b) depressive symptoms, whereas it is positively correlated with (c) the mental health component and (d) subjective well-being.

*H_2_*: The correlation between stress and mental health (i.e., depressive symptoms, mental health component, and subjective well-being) is lower among individuals with higher levels of MT.

Both hypotheses are grounded in the assumption that individuals who perceive a high level of control remain committed under adversity, view challenges as opportunity for growth, and possess strong self-belief which makes them less likely to experience stress-induced declines in mental health.

## 2 Methods

### 2.1 Participants and procedure

The present, pre-registered study^1^ was conducted in the setting of the longitudinal community-based “Gesundheit zum Mitmachen” project (for detailed information on the study design and objectives, please refer to Schmidt et al., 2022). Data collection took place between April 29 and June 07, 2025 in Bad Schönborn, Germany, as part of the project’s seventh measurement wave (previous waves: 1992, 1997, 2002, 2010, 2015, and 2021). Participants aged 33 years and older were randomly selected from the residents’ registration office and invited to participate. All participants provided written informed consent prior to data collection. The study protocol was approved by the scientific advisory council of the Schettler Clinic, Bad Schönborn, and the ethics committee of the Karlsruhe Institute of Technology (KIT), and conducted in accordance with the ethical standards of the German Psychological Society (DGPs). Participation was voluntary, and all data were analyzed anonymously.

A total of 491 individuals participated in the 2025 wave of the “Gesundheit zum Mitmachen” study. Of these, 402 participants between 34 and 90 years (211 females and 191 males; age: *M* = 57.08 years, SD = 11.80 years) completed the questionnaires relevant to the present study on stress, MT, and mental health. The majority of participants reported engaging in regular physical activity or exercise (*n* = 348), while a smaller subset of the sample currently participated in sports competitions (*n* = 54). Most participants were of German nationality (*n* = 386) and were currently employed (*n* = 266).

### 2.2 Measures

#### 2.2.1 Mental toughness

MT was assessed using the German version (VS-MTQ-G; Dziuba et al., 2025a) of the Very Short Mental Toughness Questionnaire (VS-MTQ; Kawabata et al., 2021). The VS-MTQ-G is a unidimensional measure comprising six items (e.g., “Challenges usually bring out the best in me”), which are answered on a 5-point Likert scale ranging from 1 (*strongly disagree*) to 5 (*strongly agree*). Participants were instructed to respond in accordance with their typical attitudes and behaviors, thereby capturing the trait-like nature of MT. Mean values were calculated, with higher values indicating higher levels of MT. The internal consistency of the VS-MTQ-G in this study was *ω* = 0.72.

#### 2.2.2 Perceived stress

The level of perceived stress was assessed using the German version (Schneider et al., 2020) of the 10-item Perceived Stress Scale (PSS-10; Cohen et al., 1983). The PSS-10 measures the extent to which individuals perceive their lives as being unpredictable, uncontrollable, and overwhelming. Participants rated how often they experienced certain thoughts and feelings during the past month (e.g., “In the last month, how often have you been upset because of something that happened unexpectedly?”) on a 5-point Likert scale ranging from 1 (*never*) to 5 (*very often*). Four positively worded items were reverse-coded before computing a total score, with higher values indicating higher levels of perceived stress. The PSS-10 demonstrated good internal consistency in the present study (ω = 0.86).

#### 2.2.3 Depressive symptoms

The German version (Gräfe et al., 2004) of the 9-item Patient Health Questionnaire (PHQ-9; Kroenke et al., 2001) was used to assess the severity of depressive symptoms. For each item, individuals are asked to rate how often they have experienced any of the listed problems (e.g., “Poor appetite or overeating”) over the past 7 days. Responses ranged from 0 (*not at all*) to 3 (*nearly every day*), with the sum of these scores resulting in a general index. Higher scores on this index indicate greater severity of depressive symptoms. In the present sample, the internal consistency was ω = 0.82.

#### 2.2.4 Mental health component

The mental health component is reflected by the Mental Component Summary score of the 12-item Short Form Health Survey (SF-12; Ware et al., 1996). In this study, the German version (Morfeld et al., 2011) was used. The instrument comprises 12 items representing eight health domains: physical functioning, role limitations due to physical health, bodily pain, general health, vitality, social functioning, role limitations due to emotional problems, and mental health. Response formats include Likert scales and binary options. Following standard scoring procedures outlined by Ware et al. (1995), four items were reverse-coded, indicator variables were created, and each indicator was multiplied by its respective regression weight. We calculated two summary measures, namely the Mental Component Summary (MCS) and the Physical Component Summary (PCS) scores. Both scores were subsequently transformed into norm-based scores with a mean of 50 and a standard deviation of 10. Although both summary scores were computed based on the SF-12, only the MCS was used as a primary indicator of mental health in this study, as it specifically captures mood, vitality, emotional role limitations, and general psychological functioning. The PCS, which reflects general physical health and mobility, was included as a covariate in the analyses to control for potential confounding effects of physical health on mental health outcomes. Both PCS and MCS scores range from 0 to 100, with higher scores representing better health.

#### 2.2.5 Subjective well-being

Subjective well-being was assessed using the German version (Brähler et al., 2007) of the WHO-5 Well-Being Index (Bech, 2004). The WHO-5 comprises five positively worded items (e.g., “I have felt calm and relaxed”) that capture general well-being over the past 2 weeks. Responses are given on a 6-point Likert scale ranging from 0 (*at no time*) to 5 (*all of the time*). A total score is obtained by summing all items, with higher scores indicating higher levels of well-being. The WHO-5 demonstrated good reliability in the present study (*ω* = 0.87).

### 2.3 Statistical analyses

All analyses were conducted using IBM SPSS Statistics version 29, with an alpha level of *p* < 0.05. Effect sizes were interpreted following Cohen’s (1988) conventions. Internal consistencies were evaluated using McDonald’s ω, with a threshold of ≥0.70 being considered acceptable (Dunn et al., 2014).

Descriptive statistics (*M, SD*) were computed for all study variables. Bivariate relationships between the predictor (i.e., perceived stress), the moderator (i.e., MT), and the outcome variables (i.e., depressive symptoms, mental health component, and subjective well-being) were examined using Pearson product moment correlations. In line with the pre-registered hypotheses, one-sided tests were used for directional correlations between these main study variables (i.e., negative associations between MT and perceived stress as well as depressive symptoms; positive associations between MT and mental health component as well as subjective well-being). Exploratory two-sided correlations were computed among all other study and control variables, including the physical health component (i.e., PCS), age, and gender.

To test whether MT moderates the relationship between stress and mental health, three separate moderation analyses were conducted using the PROCESS macro version 4 (Model 1; Hayes, 2022). Each model specified one mental health parameter (i.e., depressive symptoms, mental health component, or subjective well-being) as the outcome variable (Y), stress as the independent variable (X), and MT as the moderator (W). Analyses were based on 5,000 bootstrap samples with heteroscedasticity-consistent standard errors (HC4) and robust 95% bootstrap confidence intervals. Continuous predictors were mean-centered. In the case of significant interactions, simple slope analyses (at *M* ± 1 *SD*) and the Johnson–Neyman technique were applied to probe the conditional effects.

Missing data were handled using the hybrid proration–full-information approach recommended by Wu et al. (2022). When up to 30% of items were missing on a given scale, missing responses were replaced with the participant’s mean of completed items of the respective scale; otherwise, the scale score was treated as missing. For the VS-MTQ-G and WHO-5, one missing item was imputed using the respondent’s mean, whereas for the PSS-10 and PHQ-9, up to two missing items were imputed following published scoring guidelines (Kroenke et al., 2001). For the SF-12, no imputation was permitted due to its complex scoring procedure and external weighting. If any item was missing, neither the MCS nor the PCS score was calculated (Morfeld et al., 2011).

Since this is a secondary analysis within the “Gesundheit zum Mitmachen” study, no *a priori* power analysis was possible. Sample size was determined by project resources (Lakens, 2022), with an expected *N* = 400. Sensitivity analysis (G*Power 3.1; Faul et al., 2009) indicated that this sample provides 90% power with an alpha level of 0.05 to detect an effect size of *f*^2^ = 0.026 for the moderation effect in a linear multiple regression model with three predictors.

Age, gender, and the physical health component (i.e., PCS) were included as control variables, based on prior research indicating systematic associations between these variables and both MT and mental health outcomes. Specifically, previous studies have shown that MT and perceived stress may vary across age and gender, and that physical health is closely linked to both stress perception and mental health indicators (e.g., Gerber et al., 2013a; Gerber et al., 2013b; Nicholls et al., 2012). Therefore, these variables were considered relevant potential confounders that could influence the examined relationships. In case of significant correlations between these control variables and MT, moderation analyses were repeated exploratively with these variables being included as covariates.

## 3 Results

### 3.1 Association between MT, perceived stress and mental health parameters

The correlations and descriptive statistics for the main and control variables are displayed in Table 1.

**Table 1:** Correlations and descriptive statistics of all main and control variables.

Variable	1	2	3	4	5	6	7	8
1. Mental toughness	–							
2. Perceived stress	−0.45^***,a^	–						
3. Depressive symptoms	−0.37^***,a^	−0.64^***^	–					
4. Mental health component	−0.37^***,b^	−0.71^***^	−0.71^***^	–				
5. Physical health component	−0.11^***,a^	−0.18^***^	−0.21^***^	−0.03^***^	–			
6. Subjective well-being	−0.37^***,b^	−0.62^***^	−0.70^***^	−0.66^***^	−0.27^***^	–		
7. Age	−0.03^***^	−0.10^***^	−0.13^***^	−0.15^***^	−0.19^***^	0.18^***^	–	
8. Gender^c^	−0.19^***^	−0.12^***^	−0.12^***^	−0.06^***^	−0.10^***^	0.08^***^	0.12^*^	–
*M*	3.98	23.42	3.77	51.41	50.41	3.47	57.08	–
*SD*	0.46	5.62	3.31	8.25	7.65	0.84	11.80	–

^a^A one-sided negative Pearson correlation was calculated. ^b^A one-sided positive Pearson correlation was calculated. ^c^Gender was coded with 0 = female and 1 = male.*p < 0.05; **p < 0.01; ***p < 0.001.

In accordance with the first hypothesis, higher levels of MT were significantly associated with reduced levels of perceived stress and less depressive symptoms, as well as higher scores in the mental health component and increased subjective well-being. All of these correlations were of moderate size ranging from −0.45 to 0.37. These findings support the assumption that higher levels of MT are linked to better mental health and overall well-being.

Beyond the first hypothesis, we also observed that higher levels of perceived stress were associated with poorer mental health outcomes, i.e., more depressive symptoms (*r* = 0.64), lower mental and physical health component scores (*r* = −0.71 and −0.18, resp.), and reduced subjective well-being (*r* = −0.62). Furthermore, the mental health parameters themselves exhibited strong correlative patterns, with all significant associations aligning with theoretical expectations, except for a non-significant relationship between the mental and physical health component.

Small, significant correlations were observed between age and several variables (−0.03 ≤ *r* ≤ 0.18). A negative association was found between age and stress, as well as positive associations with depressive symptoms, the mental health component, and subjective well-being. There were also small correlations with regard to gender (−0.12 ≤ *r* ≤ 0.19). Males tended to report higher MT and better physical health, whereas females reported more stress and depressive symptoms.

### 3.2 Moderating effect of MT on the relationship between stress and mental health

**Figure 1:**
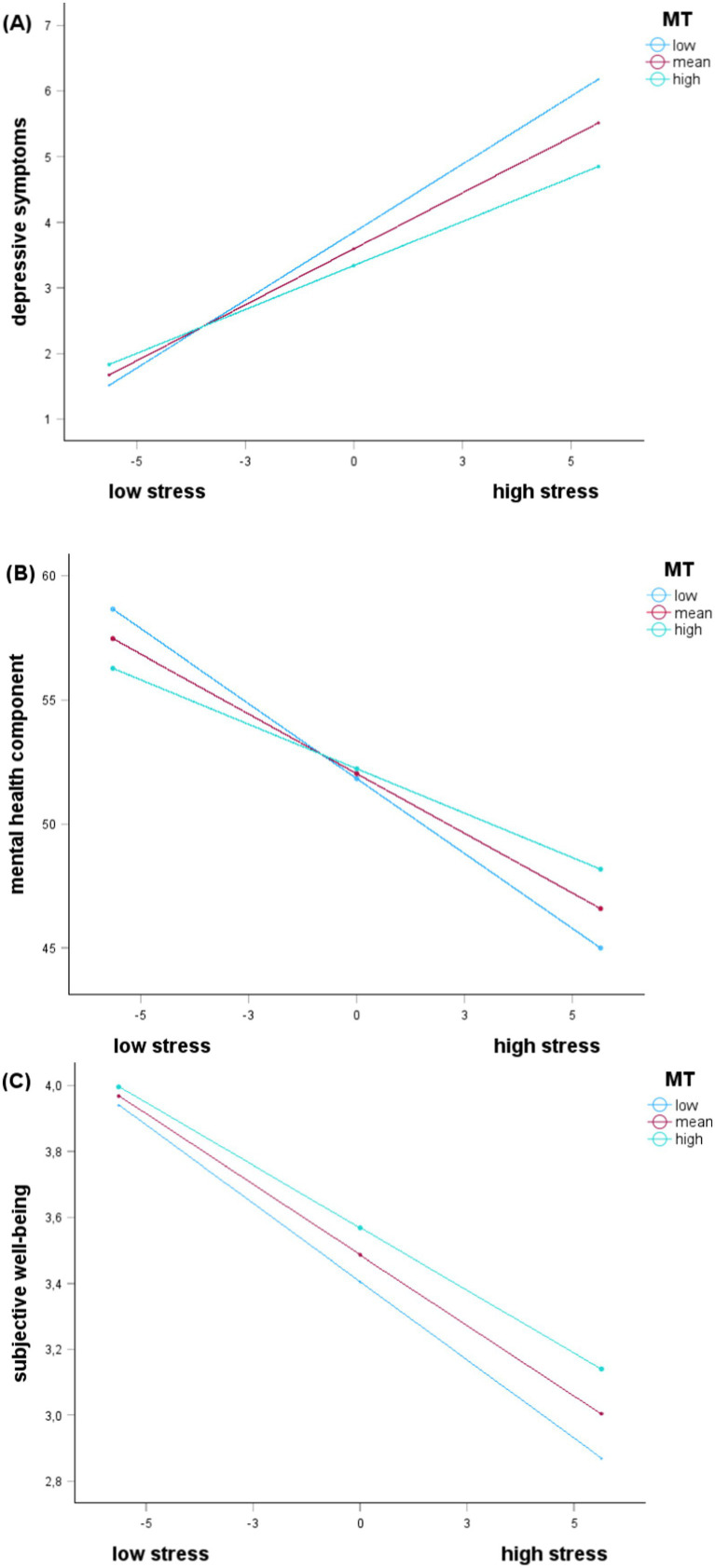
The associations between perceived stress and **(A)** depressive symptoms, **(B)** the mental health component and **(C)** subjective well-being among persons with three different levels of MT. Values for the moderator are the mean (0) and ±1SD (±0.45).

The results regarding the second hypothesis are summarized in Table 2, with the corresponding interaction plots displayed in Figures 1A–C.

**Table 2:** Moderated regression analyses for the three mental health parameters.

Outcome variable	Predictor	*b*	SE	95% CI [LL, UL]	*t*	*p*
Depressive symptoms	Stress	0.34	0.04	[0.27, 0.42]	8.83	<0.001
	MT	−0.56	0.35	[−1.24, 0.13]	−1.60	0.111
	Stress × MT	−0.16	0.06	[−0.27, −0.05]	−2.89	0.004
Mental health component	Stress	−0.96	0.07	[−1.11, −0.82]	−13.02	<0.001
	MT	0.44	0.75	[−1.03, 1.91]	0.59	0.557
	Stress × MT	0.54	0.10	[0.34, 0.74]	5.30	<0.001
Subjective well-being	Stress	−0.09	0.01	[−0.10, −0.07]	−10.94	<0.001
	MT	0.18	0.08	[0.03, 0.33]	2.35	0.020
	Stress × MT	0.02	0.01	[−0.00, 0.04]	1.84	0.067

b = Unstandardized regression coefficients. All predictors were mean-centered. Confidence intervals are based on HC4-corrected standard errors (heteroscedasticity-robust).

In a first step, as specified in the preregistration, we examined whether the planned covariates (i.e., age and the physical health component) were correlated with the moderator variable (i.e., MT). While the physical health component showed a significant association with MT and was therefore included in the follow-up analyses, age did not correlate significantly with MT and was consequently not added as a covariate. A minor deviation from the preregistered plan was the inclusion of gender as an additional covariate in a follow-up analysis, due to its significant association with MT in the present sample. The results of the analyses including the physical health component and gender as covariates are presented in ESM1.

For depressive symptoms, the overall model was significant, *F*(3, 394) = 52.33, *p* < 0.001, *R*^2^ = 0.44. Higher perceived stress was associated with more depressive symptoms (*b* = 0.34, *SE* = 0.04, *p* < 0.001). The main effect of MT was not statistically significant (*p* = 0.111). However, as anticipated, the interaction between stress and MT was statistically significant (*b* = −0.16, *SE* = 0.06, *p* = 0.004), accounting for an additional 2.3% of explained variance. Simple slope analyses indicated that stress was positively associated with depressive symptoms at all levels of MT, but this association weakened as MT increased. Consequently, individuals with higher levels of MT reported fewer depressive symptoms under comparable stress levels (Figure 1A).

For the mental health component, the model was also statistically significant, *F*(3, 390) = 90.35, *p* < 0.001, *R*^2^ = 0.54. Perceived stress was negatively correlated with the mental health component (*b* = −0.96, *SE* = 0.07, *p* < 0.001), while MT demonstrated no statistically significant main effect (*p* = 0.557). The interaction between stress and MT was statistically significant (*b* = 0.54, *SE* = 0.10, *p* < 0.001), explaining an additional 4.2% of variance. Conditional effects revealed that stress predicted worse mental health across all levels of MT, but the strength of this relationship was reduced at higher levels of MT, suggesting a moderating effect of MT (Figure 1B).

Finally, in terms of subjective well-being, the model also reached statistical significance, *F*(3,392) = 65.53, *p* < 0.001, *R*^2^ = 0.41. Perceived stress was negatively associated with subjective well-being (*b* = −0.09, *SE* = 0.01, *p* < 0.001), whereas MT exerted a positive main effect (*b* = 0.18, *SE* = 0.08, *p* = 0.020). The interaction between stress and MT was not statistically significant (*b* = 0.02, *SE* = 0.01, *p* = 0.067). Despite this, the visual display of interaction findings indicates that higher levels of MT were descriptively associated with weaker negative associations between stress and well-being (Figure 1C).

## 4 Discussion

This study yielded two key findings. First, higher levels of MT were associated with lower levels of perceived stress and better mental health (i.e., less depressive symptoms, better mental health component, and increased subjective well-being). Second, MT was found to moderate the relationship between perceived stress and both depressive symptoms and the mental health component. No significant interaction was observed for subjective well-being.

### 4.1 The relationship between MT, stress, and mental health

In line with our first hypothesis, we observed that higher levels of MT were negatively correlated with perceived stress, and positively correlated with mental health. Participants with higher levels of MT were less likely to perceive stress, and reported fewer depressive symptoms, while reporting a better mental health component and better subjective well-being.

These results are consistent with earlier research indicating that individuals with higher levels of MT tend to experience less stress (e.g., Haghighi and Gerber, 2019; Kawabata et al., 2021). Based on Clough et al.’s (2002) 6C model, mentally tough individuals are more likely to appraise potentially stressful situations as challenges rather than threats (challenge), feel capable regarding their abilities and interpersonal skills (confidence), experience a sense of control in their life circumstances and emotional reactions (control), and maintain effort toward goal achievement despite adversity (commitment). These characteristics may help to explain why individuals with higher levels of MT are less susceptible to the negative effects of stress (Levy et al., 2012; Nicholls et al., 2012), and why they tend to report lower stress intensities than individuals with lower MT when exposed to comparable stressors (Kaiseler et al., 2009). In addition, the Social Safety Theory (Slavich, 2020) offers a complementary multilevel framework for understanding stress adaptation by emphasizing how perceptions of social threat and safety may influence downstream biological responses to stress. Within this framework, individual differences in psychological traits such as MT may impact not only psychological stress appraisal processes but also biological responses to stress. Incorporating this perspective is valuable as it may help situate MT within a broader integrative model of stress perception and adaptation.

In sport contexts, MT has been associated with more effective management of physical (e.g., fatigue) and psychological (e.g., stress, pressure) demands (Gucciardi et al., 2015), which may facilitate better performance under stress (Jones et al., 2007; Weinberg, 2010). Evidence also suggests that higher levels of MT are linked to more adaptive problem-focused coping strategies, whereas lower levels of MT are more often associated with avoidant coping patterns, which may increase stress over time (Dziuba et al., 2025b; Gucciardi et al., 2015; Poulus et al., 2020). Overall, these findings support the assumption that MT is related to more adaptive appraisal and coping processes in the context of stress. The fact that mentally tough individuals experience less stress may partly explain our observed positive associations between MT and mental health (Nicholls et al., 2009). Previous studies found moderate to strong correlations between MT and mental health outcomes (Coulter et al., 2010; Papageorgiou et al., 2018). For example, Gerber et al. (2018) observed that mentally tough individuals might suffer less from mental disorders. In their study, MT was related to lower stress scores, lower burnout scores, and fewer depressive symptoms. According to Beck and Alford (2009), depression encompasses symptoms on an emotional (e.g., helplessness), motivational (e.g., withdrawal), cognitive (e.g., hopelessness), somatic (e.g., irritability) and motor level (e.g., agitation). MT may buffer these symptoms because MT’s core components (control, challenge, commitment, confidence) contradict typical depressive cognitions and behavior (Gerber et al., 2013b). Moreover, higher levels of MT have been linked to reduced anxiety and rumination (Dziuba et al., 2025a), as well as increased well-being (Lin et al., 2017) and life satisfaction (Clough et al., 2002; Dagnall et al., 2019; Gerber et al., 2013a).

Additional correlative patterns also deserve attention. In previous studies, MT has been associated with higher levels of physical activity (Gerber et al., 2012), which is known to be beneficial for mental health through both direct and indirect pathways (Fuchs et al., 1994). Sport contexts often involve structured exposure to manageable stressors and challenges, which may contribute to the development of MT attributes. Thus, MT may influence mental health both directly (e.g., stress-reducing mechanisms) and indirectly (e.g., via exercise-related pathways). Gerber et al. (2018) further found that neither training load nor years in competitive sport were related to mental health, suggesting that even small doses of physical activity may contribute to mental health.

Gender differences as observed in our study resembled previous findings with males reporting higher levels of MT (Gerber et al., 2012, 2015, 2018), lower stress scores, and more favorable mental health indicators such as fewer depressive symptoms as well as increased life satisfaction (Gerber et al., 2013a). It might be reasonable to assume that these differences may partly reflect gender-related socialization processes, whereby men are often encouraged to display confidence, emotional control, and persistence, while women tend to report stress and emotional strain more openly (Addis and Mahalik, 2003). Additionally, some studies suggest that men engage more frequently in problem-focused coping, whereas women may rely more on emotion-focused or ruminative strategies, which can heighten perceived stress and increase vulnerability to depressive symptoms (Matud, 2004; Nolen-Hoeksema, 2012).

Age-related associations showed a complex and somewhat heterogeneous pattern in our study. While age was negatively associated with perceived stress and the physical health component, it was positively related to depressive symptoms as well as to the mental health component and subjective well-being. This pattern suggests that different facets of mental health may follow distinct trajectories across the lifespan rather than changing uniformly with age. For example, age-related increases in subjective well-being may coexist with higher levels of physical complaints or specific depressive symptoms. Although the observed associations were small, they may nonetheless reflect meaningful, multidimensional age-related processes and should thus be examined in future research.

### 4.2 Moderating effect of MT on the relationship between stress and mental health

Moreover, in line with our second hypothesis, results showed that in addition to bivariate relationships, MT moderated the relationship between stress and mental health outcomes in terms of depressive symptoms and the mental health component. In contrast, the interaction between MT and stress did not reach statistical significance for subjective well-being. This pattern may point to meaningful differences in how MT relates to distinct dimensions of mental health. While depressive symptoms primarily reflect psychological distress and the mental health component captures broader aspects of psychological functioning, subjective well-being represents a positive dimension of mental health characterized by positive affect and life satisfaction (Diener et al., 2003; Westerhof and Keyes, 2010). The present findings may therefore suggest that MT is more strongly related to reducing vulnerability to distress than to maintaining and enhancing positive well-being under stress. Alternatively, other resources (e.g., social, contextual, or affective factors) may play a more prominent role in shaping subjective well-being (for an overview, see also Diener et al., 2003). Although a descriptive trend suggested a weaker decline in subjective well-being across stress levels among individuals with higher MT, the lack of statistical significance underscores the need for cautious interpretation and highlights the importance of further research to clarify these potentially differential pathways.

These results align with previous studies showing that MT can moderate the negative effects of stress among high school students, vocational students, and university students (Gerber et al., 2013a; Gerber et al., 2013b; Gerber et al., 2015) as well as elite athletes (Gerber et al., 2018). For example, MT predicted lower depressive symptoms and increased life satisfaction over a 10-month period (Gerber et al., 2013a). The current study extends the previous literature by showing that MT moderates the relationship between stress and mental health in a broader, community-based sample of adults. However, earlier work also reported mixed findings, with one study identifying stress-buffer effects of MT for burnout but not for depression in cross-sectional analyses, whereas in the prospective analyses, the interaction between MT and stress predicted both burnout and depressive symptoms over a 6-month period (Gerber et al., 2018). Furthermore, recent theoretical work suggests that MT and mental health are not contradictory, but rather mutually supportive. Gucciardi et al. (2017) argued that MT should not be understood as emotional suppression or invulnerability. Rather, MT is characterized by cognitive-emotional skills that enables individuals to cope with stress more effectively (Kaiseler et al., 2009; Nicholls et al., 2008; Petrie et al., 2014). This view is consistent with our findings, which suggest that MT is associated with better mental health without implying that individuals with high MT are unaffected by stress.

Several mechanisms may explain why MT promotes better mental health. On the one hand, studies have shown that positive associations exist between MT and psychological constructs such as optimism and self-efficacy (Clough et al., 2002; Nicholls et al., 2008). On the other hand, MT is positively associated with higher quality of sleep (Brand et al., 2010; Brand et al., 2016), and increased life satisfaction (Brand et al., 2016; Clough et al., 2002; Gerber et al., 2013a). Collectively, these findings suggest that MT functions as a resilience resource that mitigates the harmful effects of stress on psychological functioning (Gerber et al., 2018). Consistent with broader resilience research, factors such as positive attributional style, perceived competence, and self-regulatory beliefs similarly reduce vulnerability to stress-related mental health problems (Grant et al., 2006).

### 4.3 Limitations and future research directions

While the large sample size and rigorous assessment of the main psychological constructs reflect major strengths of our study, there are several limitations which need to be acknowledged. A main limiting factor that should be considered is that all data was collected through self-report instruments, which may introduce response biases (e.g., Podsakoff et al., 2003). Second, the cross-sectional design prevents causal interpretations. Longitudinal and experimental studies are thus needed to extend our knowledge regarding temporal relationships, pathways, and potential reciprocal effects between MT, stress, and mental health. At the same time, recent evidence points to possible limitations. A meta-analysis by Liang et al. (2024) reported positive associations between MT and certain dark personality traits (narcissism, Machiavellianism, and psychopathy), suggesting that MT includes both adaptive and less adaptive components depending on how it is expressed. Thus, it should be noted that while the present study emphasizes the protective aspects of MT, the broader literature highlights the importance of conceptualizing MT as a balanced construct rather than an exclusively positive trait.

A further limitation concerns the external validity of the findings. Although the sample comprised a relatively broad age range (34–90 years), younger adults were not represented. Consequently, the generalizability of the findings to earlier stages of adulthood remains unclear, as stress experiences and coping resources may differ across age groups. The present study was designed to extend previous research that has predominantly focused on adolescents, students, and athletic populations (e.g., Dziuba et al., 2025a, 2025b) by examining these associations in a broader community-based adult sample. Furthermore, while the sample covered a broad age range, it remained relatively homogeneous in several respects, including high levels of physical activity and predominantly German nationality. These characteristics may reflect a specific subgroup of the population and may have introduced selection bias. In addition, participation was voluntary, raising the possibility of self-selection effects. Together, these factors limit the representativeness of the sample and restrict the generalizability of the findings to populations with different demographic, cultural, socioeconomic, or lifestyle characteristics. Future studies should therefore seek to replicate these findings in more diverse and representative samples.

An additional limitation concerns the use of the VS-MTQ-G, a brief, unidimensional measure that captures overall MT but does not differentiate between the specific facets proposed in the multidimensional concept of MT (i.e., 6C model). The use of a brief instrument was considered appropriate in the context of the present community-based study, as it reduced participant burden while still providing a reliable global indicator of MT. However, this approach limits the ability to examine differential effects of specific facets of MT. This should be considered when interpreting the findings.

Although the moderation model applied in this study provides insights into associations between MT, stress, and mental health, the underlying mechanisms (e.g., coping or emotion regulation processes) remain unclear and warrant further investigation using longitudinal or mediation-based designs. While resilience is often understood to develop through exposure to manageable stressors rather than their avoidance (Rutter, 1993), and MT has been conceptualized as potentially modifiable across the lifespan despite genetic influences (Horsburgh et al., 2009), the specific processes through which MT may change remain unclear. Accordingly, intervention research may help clarify whether MT can be effectively enhanced and how such changes relate to mental health outcomes (e.g., Clough and Strycharczyk, 2012; Soundara Pandian et al., 2023). Also, from a translational perspective, integrating biologically informed frameworks such as Social Safety Theory (Slavich, 2020) may further guide future intervention research and provide a broader context for linking MT to downstream stress-related health effects. Overall, future work should place greater emphasis on underlying mechanisms and temporal dynamics to better understand the role of MT in stress adaptation.

## 5 Conclusion

The present study shows that MT is associated with lower stress and more favorable mental health outcomes in community-dwelling adults aged 34 years and older in a cross-sectional setting. Moreover, MT appeared to buffer the relationship between stress and mental health, particularly regarding depressive symptoms and the mental health component. These findings suggest that MT may be a relevant factor in mental health promotion and indicate a potential role in interventions aimed at supporting stress resilience.

## Data Availability

The datasets generated and analyzed for the current study are not publicly available due to the strict ethical standards as required by The Ethics Committee of the Karlsruhe Institute of Technology, Germany. However, data may be available from the corresponding author on reasonable request. Requests to access the datasets should be directed to Alexander.Woll@kit.edu.

## Footnotes

1
https://osf.io/dc8k7
