# Supplementary material for: The moderating effect of mental toughness on the relationship between stress and mental health
Source: Front Psychol. 2026 Jul 1;17:1768455. doi: 10.3389/fpsyg.2026.1768455 (PMC13370633; doi:10.3389/fpsyg.2026.1768455)
Supplement: Supplementary file 1 [file Table_1.DOCX]

Additional moderated regression analyses with covariates

To examine the robustness of the main findings, additional moderated regression analyses were conducted for all three outcome variables (i.e., depressive symptoms, mental health component, and subjective well‑being) including the physical health component and gender as covariates. As specified in the preregistration, only those covariates showing significant associations with the moderator (i.e., MT) were included. While the physical health component correlated significantly with MT and was therefore included in the analyses, age did not, so it was omitted. As a minor deviation from the preregistered plan, gender was also included due to its significant association with MT in the present sample.

The main results for the additional analyses with the covariate physical health component can be found in Table 1.

Table 1. Moderated regression analyses with physical health component as covariate

| Outcome variable | Predictor | *b* | *SE* | 95% CI [LL, UL] | *t* | *p* |
| --- | --- | --- | --- | --- | --- | --- |
| Depressive symptoms | | | | | | |
|  | stress | 0.34 | 0.04 | [0.26, 0.42] | 8.21 | < .001 |
|  | MT | –0.46 | 0.35 | [–1.14, 0.22] | –1.33 | .186 |
|  | stress × MT | –0.16 | 0.05 | [–0.27, –0.06] | –2.99 | .003 |
|  | covariate | –0.04 | 0.02 | [–0.09, 0.00] | –1.86 | .064 |
| Mental health component | | | | | | |
|  | stress | –1.00 | 0.07 | [–1.15, –0.85] | –13.37 | < .001 |
|  | MT | 0.56 | 0.72 | [–0.86, 1.98] | 0.78 | .439 |
|  | stress × MT | 0.54 | 0.09 | [0.36, 0.72] | 5.84 | < .001 |
|  | covariate | –0.18 | 0.05 | [–0.27, –0.08] | –3.59 | < .001 |
| Subjective well-being | | | | | | |
|  | stress | –0.08 | 0.01 | [–0.10, –0.07] | –10.23 | < .001 |
|  | MT | 0.17 | 0.08 | [0.01, 0.32] | 2.16 | .032 |
|  | stress × MT | 0.02 | 0.01 | [–0.00, 0.04] | 1.83 | .068 |
|  | covariate | 0.02 | 0.01 | [0.00, 0.03] | 2.69 | .008 |

*Notes:* *b* = unstandardized regression coefficients. All predictors were mean-centered. Confidence intervals are based on HC4‑corrected standard errors (heteroscedasticity-robust).

Across all models, the inclusion of the physical health component led to only marginal changes in the regression parameters, confirming the stability of the main effects. Specifically, the interaction between perceived stress and MT remained significant for both depressive symptoms (*b* = –0.16, *SE* = 0.05, *p* = .003) and the mental health component (*b* = 0.54, *SE* = 0.09, *p* < .001) and yielded similar results for subjective well‑being (*b* = 0.02, *SE* = 0.01, *p* = .068). The physical health component contributed modestly to explained variance (ΔR² ≈ .02–.03), primarily through negative association with the mental health component (*b* = –0.18, *SE* = 0.05, *p* < .001) and positive association with subjective well‑being (*b* = 0.02, *SE* = 0.01, *p* = .008), but not with depressive symptoms (*b* = –0.04, *SE* = 0.02, *p* = .064).

The main results for the additional analyses with the covariate gender can be found in Table 2.

Table 2. Moderated regression analyses with gender as covariate

| Outcome variable | Predictor | *b* | *SE* | 95% CI [LL, UL] | *t* | *p* |
| --- | --- | --- | --- | --- | --- | --- |
| Depressive symptoms | | | | | | |
|  | stress | 0.34 | 0.04 | [0.26, 0.42] | 8.83 | < .001 |
|  | MT | –0.51 | 0.36 | [–1.21, 0.19] | –1.44 | .150 |
|  | stress × MT | –0.16 | 0.06 | [–0.27, –0.05] | –2.88 | .004 |
|  | covariate | –0.24 | 0.26 | [–0.75, 0.26] | –0.95 | .343 |
| Mental health component | | | | | | |
|  | stress | –0.96 | 0.07 | [–1.11, –0.82] | –12.99 | < .001 |
|  | MT | 0.53 | 0.76 | [–0.96, 2.02] | 0.70 | .483 |
|  | stress × MT | 0.54 | 0.10 | [0.34, 0.74] | 5.24 | < .001 |
|  | covariate | –0.50 | 0.57 | [–1.62, –0.62] | –0.87 | .382 |
| Subjective well-being | | | | | | |
|  | stress | –0.09 | 0.01 | [–0.10, –0.07] | –10.91 | < .001 |
|  | MT | 0.19 | 0.08 | [0.03, 0.34] | 2.39 | .017 |
|  | stress × MT | 0.02 | 0.01 | [–0.00, 0.04] | 1.83 | .068 |
|  | covariate | –0.02 | 0.07 | [–0.15, 0.11] | –0.34 | .735 |

*Notes:* *b* = unstandardized regression coefficients. All predictors were mean-centered. Confidence intervals are based on HC4‑corrected standard errors (heteroscedasticity-robust).

Across all models, the inclusion of gender produced no meaningful changes in the regression parameters or significance levels, confirming the stability of the main results. The interaction between perceived stress and MT remained significant for both depressive symptoms (*b*= –0.16, *SE* = 0.06, *p* = .004) and the mental health component (*b* = 0.54, *SE* = 0.10, *p* < .001) and yielded similar results for subjective well-being (*b* = 0.02, *SE* = 0.01, *p* = .068). Gender itself was not a significant predictor in any model (.343 ≤ *p* ≤ .735).

Overall, the additional analyses support the main findings. Including the physical health component produced only minor changes in coefficients and did not alter the significance of the interaction between stress and MT for any outcome. Although the physical health component showed small associations with the mental health component and subjective well‑being, it explained only limited additional variance and did not account for the core moderation effect. Likewise, gender was not a significant predictor and did not influence the strength or significance of the interaction.

Taken together, these results indicate that the stress-buffering role of MT on depressive symptoms and the mental health component is stable across models and largely independent of gender and the physical health component, underscoring MT as a general psychological resilience factor.
